# Prognostic impact of mRNA levels of LGR5 transcript variants in OSCC patients

**DOI:** 10.1186/s12885-019-5327-8

**Published:** 2019-02-15

**Authors:** Swetlana Rot, Tom Kaune, Helge Taubert, Thomas Greither, Johanna Kotrba, Antje Güttler, Henri Wichmann, Udo Bilkenroth, Andreas Wienke, Bilal Al-Nawas, Matthias Bache, Dirk Vordermark, Claudia Wickenhauser, Daniel Bethmann, Alexander W. Eckert, Matthias Kappler

**Affiliations:** 1 Department of Oral and Maxillofacial Plastic Surgery, Martin Luther University Halle-Wittenberg, Ernst-Grube-Str, 40 06097 Halle/Saale, Germany; 2 Clinic of Urology and Pediatric Urology, FA University Hospital Erlangen, FA University Erlangen-Nürnberg, Erlangen, Germany; 3 Centre for Reproductive Medicine and Andrology, Martin Luther University Halle-Wittenberg, Halle (Saale), Germany; 4 Department of Radiotherapy, Martin Luther University Halle-Wittenberg, Halle (Saale), Germany; 5 Institute of Pathology, Eisleben, Germany; 6 Institute of Medical Epidemiology, Biostatistics, and Informatics, Martin Luther University Halle-Wittenberg, Halle (Saale), Germany; 7 Institute of Pathology, Martin Luther University Halle-Wittenberg, Halle (Saale), Germany; 8 Present address: Department of Internal Medicine I, Martin Luther University Halle-Wittenberg, Halle (Saale), Germany; 9 Present address: Institute of Molecular and Clinical Immunology, Otto-von-Guericke-University, Magdeburg, Germany; 10 Present address: Department of Oral and Maxillofacial Surgery, Plastic Surgery, University Medical Center of the Johannes Gutenberg-University Mainz, Mainz, Germany

**Keywords:** Head and neck squamous cell carcinoma, overall survival, stem cell-associated gene, LGR5, Splice variants, EMT

## Abstract

**Background:**

The human leucine-rich, repeat-containing G protein-coupled receptor 5 (*LGR5*) is a stem cell marker in numerous adult tissues and is overexpressed in a large number of human carcinoma including colon cancer, breast cancer and oral squamous cell carcinomas (OSCC). The role of the full length transcript (*LGR5FL*) in progression and prognosis of several cancers was reported. However, the biological function of three splice variants of *LGR5* (*LGR5Δ5*, *LGR5Δ8* and *LGR5Δ5–8*) has yet to be thoroughly investigated.

**Methods:**

Seventy-eight frozen tumor samples from adult OSCC patients were studied using quantitative real-time TaqMan™ PCR analysis. The mRNA levels of full length *LGR5*, the splice variant of *LGR5* lacking exon 5 (*LGR5Δ5*), the splice variant of *LGR5* lacking exon 8 (*LGR5Δ8*) and the mRNA level of all known transcript variants together (*LGR5all*) were quantified and correlated to overall and disease-specific survival of OSCC patients, clinical parameters and the mRNA level of different tumor-associated markers.

**Results:**

An elevated level of tumoral *LGR5Δ5* mRNA, but not *LGR5FL, LGR5Δ8* or *LGR5all* mRNA was significantly associated with a poor prognosis for the overall and disease-specific survival of OSCC patients (hazard ratio (HR) = 2.0; *p* = 0.02; 95% CI: 1.1–3.7; HR = 3.2; *p* = 0.01; 95% CI: 1.3–8.0; multivariable Cox regression), respectively. Additionally, a higher tumoral level of *LGR5Δ5* mRNA in primary tumors was associated with the occurrence of regional lymph node metastases in OSCC patients (odds ratio (OR) = 3.1; *p* = 0.022; 95% CI: 1.2–7.9; binary logistic regression). Furthermore, the mRNA levels of all investigated *LGR5* transcript variants were significantly correlated with the mRNA expression of Wnt-target genes and markers of epithelial-to-mesenchymal transition (EMT).

**Conclusion:**

The mRNA level of the *LGR5* splice variant *LGR5Δ5* is an independent negative prognostic marker for overall and disease-specific survival and metastasis in OSCC patients. Additionally, we suggest, all *LGR5* transcript variants are involved in the EMT process mainly through activating the Wnt-signalling pathway.

## Background

Cancer of the lip and oral cavity represents the 15th most common cancer worldwide with 410,304 new cases and 146,000 deaths in 2015 [1]. Over 90% of all malignancies in the oral cavity are squamous cell carcinomas (SCC) [2]. Although diagnosis at earlier stage improved outcome of the patients in the last decades the 5-years survival rate of OSCC patients has stagnated at approximately 40–50% despite the advances in the therapeutic techniques [3–5]. Therefore, new therapeutic strategies are needed in order to improve the patient’s recurrence rates and the overall survival and therefore independent molecular biomarkers are necessary which help to estimate the prognosis and the efficacy of an individual therapeutic strategy.

As other cancers, OSCCs show a heterogeneity in their cellular morphology [6]. In general, two models have been established in order to explain the underlying mechanisms of tumor heterogeneity: I) the clonal evolution model and II) the cancer stem cell (CSC) hypothesis [7–9]. At this moment a model where the CSC hypothesis is integrated into the clonal evolution model is favoured. This model postulates that genetically distinct tumor subclones harbour subpopulations of different tumor initiating cells (CSC). These CSCs are capable of self-renewal and drive tumor growth, recurrence and metastasis as well as the resistance to therapeutic approaches [10]. Therefore, it is necessary to introduce therapies that target not only the rapidly proliferating tumor cells of the tumor mass, but especially the tumor initiating cells in order to avoid therapeutic failures [11]. New molecular markers which accurately identify CSC cells are essential for those new therapeutic options.

One such candidate molecular marker is the leucine-rich repeat-containing G protein-coupled receptor *LGR5*, a member of the G-protein-coupled receptor family of proteins and a target of Wnt signalling [12]. It was initially identified as a marker of murine small intestinal and colon stem cells [13]. *LGR5* was found to be overexpressed in colorectal cancer [14–18] and several studies indicated that *LGR5* expression is associated with colorectal carcinogenesis, tumor growth and metastasis [18–20]. Subsequent studies demonstrated that *LGR5* is expressed by a diverse range of adult tissues and organs and acts as a biomarker for adult stem cells in certain tissues including oral tissues [21, 22]. Furthermore, *LGR5* was found to be overexpressed in several carcinomas having a close association with initiation and recurrence of different cancer types and correlating with tumor growth, invasion and poor prognosis [18, 20, 23, 24].

Functionally, *LGR5* is a part of Wnt signalling complex on the cell membrane, where it appears to be able to enhance the activity of the Wnt/ß-catenin signalling [12]. Thus, LGR5 is a target gene of Wnt signalling, but because of its function also an enhancer of this Wnt signalling in the sense of a positive feedback loop. To our knowledge, three transcript variants of *LGR5* have been described until now: one lacking exons 5–8 (*LGR5Δ5*–8) as introduced by Osawa et al. [25], the second lacking exon 8 (https://www.uniprot.org/uniprot/O75473) and the third lacking exon 5 (*LGR5Δ5*), which has been previously described by our group [26]. All three variants have a truncated ligand binding domain [27]. In LGR5FL this ligand binding domain interacts with R-Spondins 1–4 resulting in the formation and internalization of a LGR5-RSPO-RNF43 protein complex, leading to a RNF43 membrane clearance, which results in a higher Wnt-activity. However, the functional effects of the truncated ligand binding domain of the LGR5 transcript variants have not been investigated yet.

Recently, the LGR5 protein expression was investigated by immunohistochemistry (IHC) in OSCC, demonstrating an increased LGR5 expression correlating with disease severity but not with patient’s outcome [22]. In a previous work our group demonstrated the mRNA expression of the *LGR5* transcript variant *LGR5Δ5*, but not the expression of full length *LGR5* (*LGR5FL*) being an independent unfavourable prognostic marker for soft tissue sarcoma patients (STS) [26]. Though to date it is not possible to differentiate between the LGR5 isoforms by IHC due to the lack of specific antibodies we were interested whether the transcript variants of LGR5 (*LGR5Δ5* or *LGR5Δ8)* in specific may affect the outcome of OSCC patients.

Therefore, we separately measured the mRNA level of *LGR5FL*, *LGR5Δ5*, *LGR5Δ8* and of all four known *LGR5* variants together (*LGR5all*) in 78 OSCC samples and correlated them with clinical parameters and the outcome of those patients.

## Methods

### Tissue samples, histomorphological data and study approval

Frozen primary tumor samples of 78 OSCC patients were analysed using the real-time quantitative PCR (qRT-PCR) analysis. All patients had been treated with surgery at the Department of Oral and Maxillofacial Plastic Surgery, Martin Luther University Halle-Wittenberg, Germany. The tissue samples were cut by a cryocut microtome and the first and the last histologic sections were stained with hematoxylin and eosin. Experienced pathologists (UB, DB) verified the sections. We defined samples as tumor tissue when > 70% of the first and the last histologic sections were tumor tissue. The clinical and histomorphological parameters of OSCC patients are shown in Table 1. The patients’ median age at the time of the diagnosis was 58.5 years (ranging from 25 to 90 years). Forty-nine OSCC patients (63%) died after an average time of 15.4 months (ranging from 0 to 56 months), and 29 OSCC patients (37%) were still alive after an average follow-up time of 44.9 months (ranging from 0 to 81 months). The study was carried out in compliance with the Helsinki Declaration, and it was approved by the Ethics Committee of the Medical Faculty of the University Halle (Ethical registry 210/19.08.09/10). All patients gave written informed consent (Department of Oral and Maxillofacial Plastic Surgery, University of Halle-Wittenberg, Germany).

**Table 1:** Clinical, histopathological and survival data

Parameters	Total (*n* = 78)	relative LGR5FL mRNA-level ≤134.3 > 134.3		relative LGR5Δ5 mRNA-level ≤2,9 > 2,9		relative LGR5Δ8 mRNA-level ≤14.9 > 14.9		relative LGR5all mRNA-level ≤8449.3 > 8449.3	
		39	39	39	39	39	39	39	39
Gender		*p* = 0.15		*p* = 0.15		*p* = 0.15		*p* = 0.15	
Male	63	29	34	29	34	29	34	29	34
Female	15	10	5	10	5	10	5	10	5
Tumor grade		*p* = 0.54		*p* = 0.58		*p* = 0.027*		*p* = 0.20	
I	9	6	3	4	5	7	2	4	5
II	57	27	30	30	27	29	28	32	25
III	11	5	6	4	7	2	9	3	8
unknown	1	1	0	1	0	1	0	0	1
Tumor stage		*p* = 0.77		*p* = 0.43		*p* = 0.36		*p* = 0.25	
I	13	8	5	8	5	6	7	9	4
II	25	13	12	14	11	12	13	11	14
III	9	4	5	5	4	7	2	6	3
IV	31	14	17	12	19	14	17	13	18
Patients at last follow-up		*p* = 0.48		*p* = 0.035*		*p* = 0.81		*p* = 0.48	
Alive	29	16	13	19	10	14	15	16	13
Dead	49	23	26	20	29	25	24	23	26
Recurrence		*p* = 0.289		*p* = 0.933		*p* = 0.289		*p* = 0.464	
Yes	25	15	10	13	12	15	10	14	11
No	51	24	27	26	25	24	27	24	27
unknown	2	0	2	0	2	0	2	1	1
Lymph node status		*p* = 0.06		*p* = 0.02*		*p* = 0.64		*p* = 0.06	
N0	30	19	11	20	10	16	14	19	11
N1–3	48	20	28	19	29	23	25	20	28
Distant metastases		*p* = 0.3		*p* = 0.3		*p* = 0.3		*p* = 1.0	
M0	74	38	36	38	36	36	38	37	37
M1	4	1	3	1	3	3	1	2	2
Survival analysis: Overall survival									
Kaplan-Meier analysis		*p* = 0.21		*p* = 0.004*		*p* = 0.88		*p* = 0.063	
Median survival (months)		27	17	42	14	25	19	42	15
95% CI		6.0–48.0	12.5–21.5	22.3–61.7	5.6–22.4	15.2–34.8	8.5–29.5	22.4–61.6	8–22.0
Univariable Cox regression		*p* = 0.22		*p* = 0.005*		*p* = 0.88		*p* = 0.069	
Hazard ratio		1.4		2.3		1.1		1.7	
95% CI		0.8–2.5		1.3–4.0		0.6–1.8		0.9–3.0	
Multivariable Cox regression		*p* = 0.2		*p* = 0.02*		*p* = 0.66		*p* = 0.09	
Hazard ratio		1.5		2.0		1.1		1.7	
95% CI		0.8–2.7		1.1–3.7		0.6–2.1		0.9–3.0	
Survival analysis: Disease specific survival									
Kaplan-Meier analysis		*p* = 0.22		*p* = 0.001*		*p* = 0.40		*p* = 0.018	
Median survival (months)		n.c.	17	n.c.	14	n.c.	19	n.c.	15
95% CI			14.0–88		0–47		7.8–104		0.8–111
Univariable Cox regression		*p* = 0.22		*p* = 0.002*		*p* = 0.4		*p* = 0.023*	
Hazard ratio		1.6		3.7		1.38		2.5	
95% CI		0.75–3.4		1.6–8.6		0.65–2.95		1.13–5.5	
Multivariable Cox regression		*p* = 0.31		*p* = 0.01*		*p* = 0.57		*p* = 0.071	
Hazard ratio		1.5		3.2		1.29		2.2	
95% CI		0.67–3.6		1.3–8.0		0.54–3.1		0.94–5.0	

*p-value ≤0.05 indicates statistical significance. n.c.- not calculable

### Quantitative RT-PCR

Total RNA of the frozen tissue samples was extracted using the Trizol reagent (Invitrogen, Karlsruhe, Germany) and 6 μg was used to synthesize cDNA using the RevertAid™ H Minus First Strand cDNA Synthesis Kit (Fermentas, St.Leon-Rot,Germany) according to manufacturer’s instructions. The mean 260/280 value over all samples was determined to be 1.93 (standard deviation SD = 0.06), the mean 230/260 to be 2.04 (SD = 0.16). Real-time quantitative PCR analysis (qRT-PCR) was performed in duplicate on a Rotorgene RG-6000 (LTF, Wasserburg, Germany) using TaqMan™ assays (ABI) for *LGR5all* (including all known *LGR5* transcript variants) *OPN*, *MMP7*, *TWIST1*, *NANOG*, *Oct3/4*, *SNAI1*, *P4HA1*, *ZEB2*, *TGFβ*, *CTGF*, *RSPO1*, *RNF43*, *IGF2*, *Vimentin* and *RPII*, which was used as endogenous control.

qRT-PCR reactions for *LGR5FL, LGR5Δ5* and *LGR5Δ8* transcript variants were performed using the Biozym Blue Probe qPCR Mix (Biozym) according to manufacturer’s instructions and the primer/probe sets: *LGR5FL* primer forward 5`-AAACCTCTCCAGCTTGGTAG-3`, primer reverse 5`-CGACCTGATATTGTTGCTATGAAATC-3`, probe 5`-FAM-CCTGGGAAAGAAATGCTTTGATGGGC-BHQ1–3`; *LGR5Δ5* primer forward 5`-GCCTTCAATCCCTACATTTC-3`, primer reverse 5`-CGACCTGATATTGTTGCTATGAAATC-3`, probe 5`-FAM-CCTGGGAAAGAAATGCTTTGATGGGC-BHQ1–3`; *LGR5Δ8* primer forward 5`-CCAACCTTAAAGAACTACATTTC-3`, primer reverse 5`-AGGTAAATGTTGAAAAGCAG-3`, probe 5`-FAM-TGACAATCCCATCCAGTTTGTTGG-MGB-3`.

The results were normalized to *RPII* transcripts amount and expressed as ΔΔCt [28]. For the analysis the patients cohort was subdivided in two groups according to the LGR5FL, LGR5Δ5, LGR5Δ8 and LGR5all median mRNA levels. An elevated expression of *LGR5FL* was determined as a median relative transcript level of > 134.3 *LGR5FL* mRNA / *RPII* mRNA, of *LGR5Δ5* as a median relative transcript level of > 2.9 *LGR5Δ5* mRNA / *RPII* mRNA, of *LGR5Δ8* as a median relative transcript level of > 14.9 *LGR5Δ8* mRNA / *RPII* mRNA and of *LGR5all* as a median relative transcript level of > 8449 relative *LGR5all* mRNA level/relative RPII mRNA level.

### LGR5 immunohistochemistry

For immunohistochemistry (IHC), the LGR5 mAb LS-C105455 (LifespanBioscience) was used. Tissue samples were deparaffinized with xylol and transferred via alcohol into aqua dest (Elix 5 Filter System, Merck-Millipore). Antigen decloaking was performed by steaming the slides with a preheated T-EDTA buffer (ZUC029–500, 1:10 dissolved, Zytomed Systems) at pH 6.0 and 98 °C for 30 min in an oven (Braun, type 3216). After cooling down for 20 min and rinsing with aqua dest, slides were blocked for 7–10 min with 3% H_2_O_2_. Following another rinsing step and application of washing buffer (ZUC202–2500, 1:20 solution, Zytochem Plus HRP Kit / Plus Polymer System, Zytomed) the LGR-5 mAb at a dilution of 1:400 was added dropwise on the tissue area and incubated for 30 min at room temperature (RT). Following a washing step, the slides were incubated with a biotinylated secondary antibody (Broad Spectrum, Zytochem Plus HRP Kit, Zytomed) for 15 min at room temperature, rinsed with washing buffer followed by 15 min of incubation with horse radish peroxidase (HRP; Zytochem Plus HRP, Zytomed). The epitopes were visualized with DAB (10 min of DAB Substrate Kit, Zytomed). After further rinsing steps (aqua dest.), the slides were counterstained with hemalaun (Dr. K. Hollborn & Sons) for 30 s, rinsed in water for 10 min, then transferred via alcohol into xylol and finally cover-slipped (Eukitt, ORSAtec) for bright field analysis.

### Statistical analyses

The association between the *LGR5FL*, *LGR5*∆5, *LGR5∆8* and *LGR5all* expression level and clinicopathological parameters was analysed by χ^2^ – test. The association between overall and disease-specific survival and *LGR5FL*, *LGR5Δ5*, *LGR5∆8* und *LGR5all* transcript variants mRNA levels was analysed by the log-rank test. Survival statistics were performed employing a multivariable Cox proportional hazard regression adjusted for gender, tumor staging, tumor grading and regional lymph node metastases. Receiver operating characteristic (ROC) curves, area under the curve (AUC) of the ROC and the cut-off point were calculated to determine the impact of tumoral mRNA levels of *LGR5FL*, *LGR5Δ5*, *LGR5∆8* und *LGR5all* transcript variants in patients with lymph node metastases versus patients without lymph node metastases. The association between the tumoral mRNA level of *LGR5Δ5* and the occurrence of lymph node metastases was tested by binary logistic regression with reporting of odds ratio (OR). Spearman’s correlation was used to assess the association between the mRNA levels of *LGR5FL*, *LGR5Δ5*, *LGR5∆8* und *LGR5all* transcript variants and a panel of tumor-associated markers which were analysed from the same RNA sample. Significance was defined by a *p* value of less than 0.05. For Spearman’s correlation Bonferroni corrected significance level (0.05/15) was used to cater for multiple comparisons. The follow-up time was calculated from the day of diagnosis until the day of last follow-up. The overall survival time and the disease-specific survival time were calculated from the day of diagnosis until the time of death (any reason) or until time of disease specific death of the patients.

## Results

### Expression of LGR5Δ5 but not LGR5FL or LGR5Δ8 is associated with poor clinical outcome in OSCC patients

**Fig. 1:**
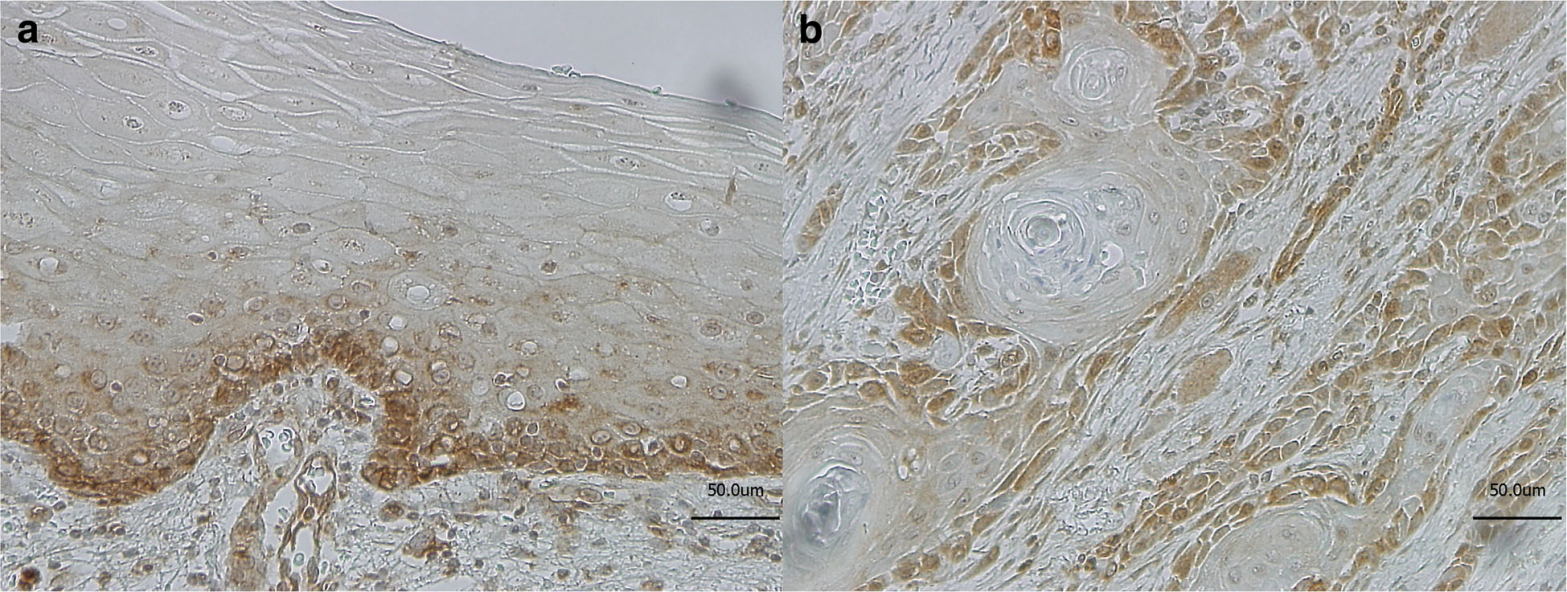
Immunostaining showing expression of LGR5 examplary in normal oral mucosa, (morphologically normal, tumor adjacent mucosa) (**a**) and in OSCC (**b**)

The expression of LGR5 in normal, dysplasia-free oral mucosa (Fig. 1a) and OSCC (Fig. 1b) was analysed by immunohistochemistry. In normal oral mucosa the LGR5 expression was restricted to the *stratum basale*. In the neoplastic epithelium of the OSCCs the LGR5 expression was diffusely expressed throughout the tumor mass except the keratinized central areas. A differentiation between the LGR5 isoforms originating from the different transcript variants by immunostaining was not possible due to the lack of specific antibodies.

For the survival analysis, the mRNA levels of *LGR5all*, *LGR5FL* and the *LGR5* transcript variants *LGR5Δ5* and *LGR5Δ8* were analysed in 78 OSCC samples and the OSCC patients’ cohort was separated into two cohorts (high and low) based on the median expression level of the *LGR5all*, *LGR5FL*, *LGR5Δ5* and *LGR5Δ8* mRNA. The median relative *LGR5FL* mRNA level of 78 OSCC samples was 134.3 (ranging from 0.5–5021; mean 391.4); the median relative *LGR5Δ5* mRNA level was 2.9 (ranging from 0 to 254.9; mean 15.01); the median relative *LGR5Δ8* mRNA level was 14.9 (ranging from 0 to 400; mean 54.3) and the median relative *LGR5all* mRNA level was 8449 (ranging from 546.4–310,418; mean 30,059), respectively.

**Fig. 2:**
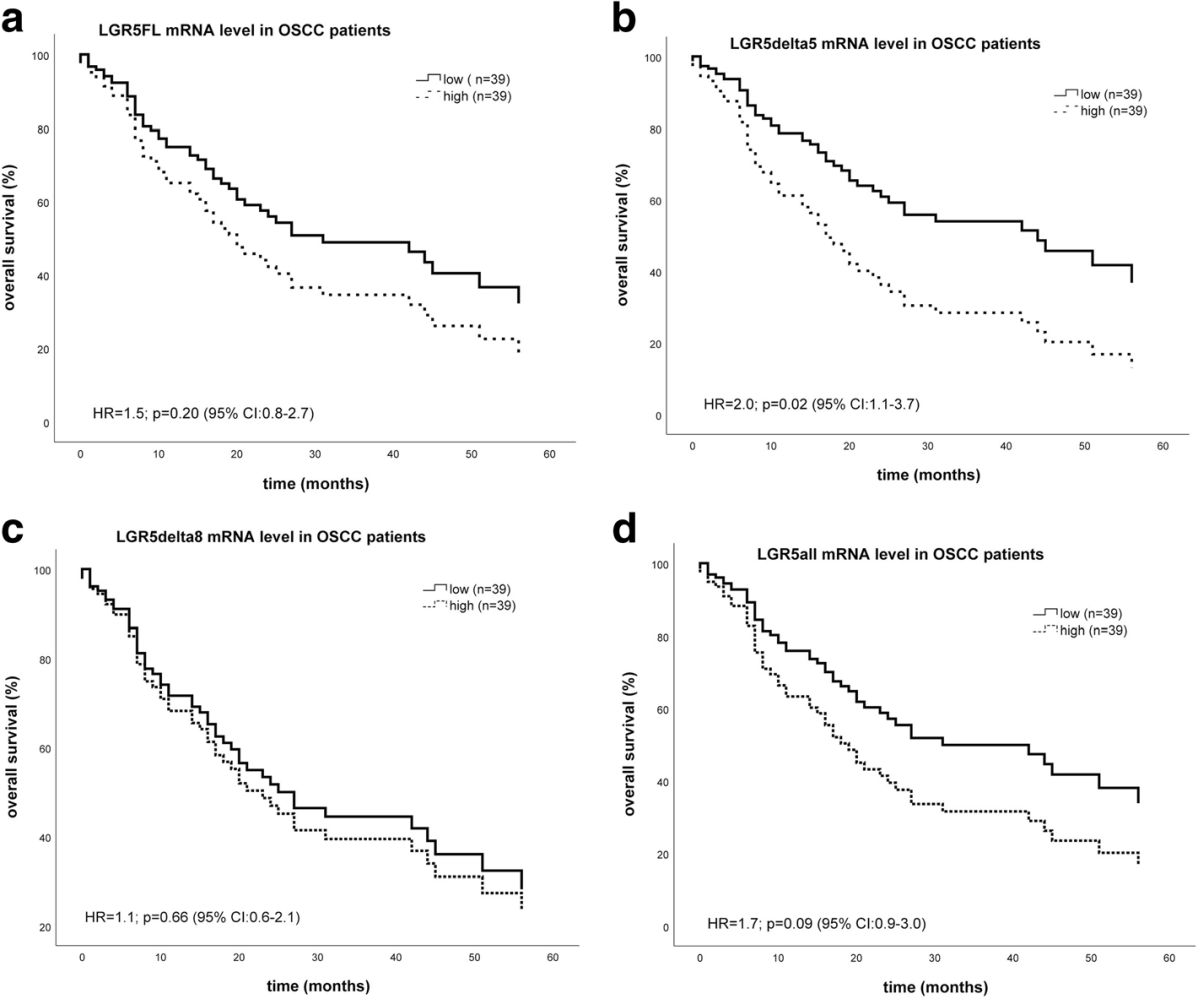
Multivariable Cox hazard regression for *LGR5FL* (**a**), *LGR5Δ5* (**b**), *LGR5Δ8* (**c**) and *LGR5all* (**d**) mRNA expression level and overall survival in OSCC patients. Relative expression level of *LGR5FL*, *LGR5Δ5, LGR5Δ8* or *LGR5all* mRNA in 78 OSCC tumor samples was correlated with overall survival. Regarding confounding factors, the Cox model was adjusted to patients’ gender, tumor stage, tumor grading and the occurrence of regional lymph node metastases. The high and low cut-off values for: **a**. *LGR5FL* were > 134.3 and ≤ 134.3 *LGR5FL* mRNA level (HR = 1.5, *p* = 0.2; CI: 0.8–2.7). **b**. *LGR5Δ5* were > 2.9 and ≤ 2.9 *LGR5Δ5* mRNA level (HR = 2.0, *p* = 0.02; CI: 1.1–3.7). **c**. *LGR5Δ8* were > 14.9 and ≤ 14.9 *LGR5Δ8* mRNA level (HR = 1.1, *p* = 0.66; CI: 0.6–2.1). **d**. *LGR5all* were > 8449.3 and ≤ 8449.3 *LGR5all* mRNA level (HR = 1.7, *p* = 0.09; CI: 0.9–3.0)

Kaplan-Meier analysis revealed a significant correlation of *LGR5∆5* mRNA expression with overall survival. OSCC patients with high tumoral mRNA levels of *LGR5Δ5* died on median 28 months earlier (median 14 +/− 8.4 months) as compared to patients with lower tumoral mRNA levels of *LGR5Δ5* (median 42 +/− 19.7 months) (*p* = 0.004) (Table 1). Multivariable Cox proportional hazard regression (confounding factors: gender, staging, grading and regional lymph node metastases) revealed that *LGR5Δ5* mRNA level was an independent prognostic factor (*p* = 0.02) for overall survival with hazard ratio of 2.0 (95% CI: 1.1–3.7) (Table1; Fig. 2b). The mRNA level of *LGR5FL, LGR5Δ8* or *LGR5all* was not associated with overall survival of OSCC patients (Table 1; Fig. 2 a, c, d). The disease-specific survival is associated with the expression of variant LGR5Δ5 in univariable and multivariable Cox analyses and the data regarding the survival analysis are recorded in Table 3.

### LGR5Δ5 expression in OSCC is correlated with the occurrence of lymph node metastases

**Fig. 3:**
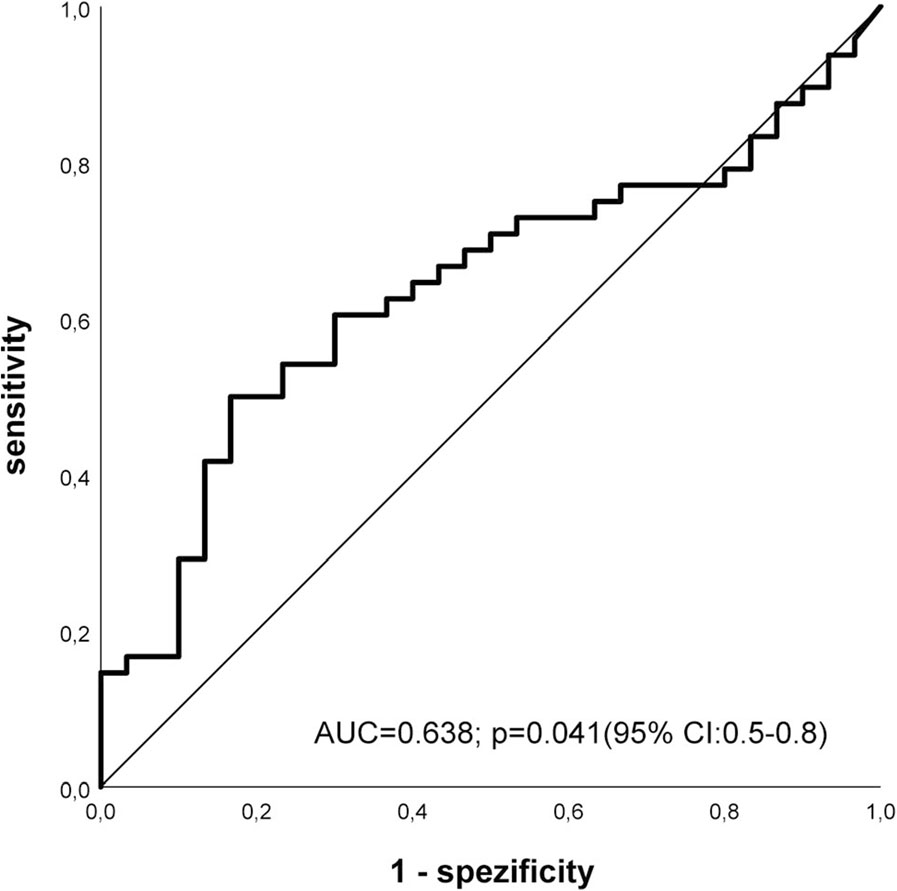
ROC curve demonstrating the sensitivity and specificity of the *LGR5*∆5 intratumoral mRNA level of patients who developed regional lymph node metastases compared to patients without metastasis. The cut-off point was 4.2 relative *LGR5Δ5* mRNA level with a corresponding AUC (area under the curve) = 63.8% (*p* = 0.041; 95% CI: 0.5–0.8).

The association between *LGR5FL*, *LGR5∆5, LGR5∆8* and *LGR5all* mRNA levels and clinicopathological parameters was analysed by χ^2^ – test and results are summarized in Table 1. OSCC cases were subdivided in two groups according to the *LGR5FL*, *LGR5Δ5*, *LGR5Δ8* and *LGR5all* median mRNA levels (high and low). High *LGR5*∆5 mRNA level was found to correlate with the occurrence of lymph node metastases with an odds ratio (OD) of 3.1 (*p* = 0.022; 95% CI: 1.2–7.9) whereas a high *LGR5*∆8 mRNA level is associated with a higher tumor grade (*p* = 0.027) (Table 1). In these cases with lymph node involvement (*n* = 48) the median level of *LGR5*∆5 mRNA in the primary lesions was 2-fold higher (4.05 vs. 1.99) when compared with those cases without lymph node metastasis (*n* = 30). For further analysis of the association between *LGR5∆5* mRNA level within the primary tumor lesions and the involvement of regional lymph nodes, ROC curves were constructed by calculating the sensitivities and specificities of the *LGR5∆5* mRNA levels to distinguish between the primary tumors with and without lymph node metastasis The cut-off point was 4.2 relative *LGR5Δ5* mRNA level (determined by the highest Youden value) at the sensitivity of 50% and a specificity of 83% with a corresponding AUC (area under the curve) of 63.8% (*p* = 0.041; 95% CI: 51.5–76.2%) (Fig. 3). In contrary to these data, the mRNA levels of *LGR5FL, LGR5Δ8* or *LGR5all* within the primary carcinoma was not associated with the occurrence of lymph node metastases (Table 1).

### LGR5 transcript variants expression correlates with the expression of Wnt-target genes involved in EMT

Analyses according to Spearman-Rho (Table 2) revealed after Bonferroni correction a significant correlation between *LGR5* transcript variants mRNA expression and the mRNA expression of tumor-associated markers. The mRNA levels of all LGR5 transcripts (*LGR5FL*, *LGR5Δ5*, *LGR5Δ8* and *LGR5all*) were positively associated with the mRNA level of *Vimentin*. The *LGR5FL*, *LGR5all* and *LGR5Δ5* mRNA levels were found to be positively correlated with *TCF-7* mRNA*.* Additionally the mRNA level of *LGR5FL* was positively associated with the *TWIST1, ZEB2, MMP7, TGFß, P4HA1, CTGF* and *IGF2* mRNA. While the *LGR5all* mRNA correlated with the mRNA level of *TWIST1*, *MMP7* and *NANOG*. Furthermore, the mRNA level of *LGR5Δ5* correlated positively with the *ZEB2, CTGF* and *IGF2* mRNA. Moreover, *LGR5Δ8* mRNA level is positively associated with mRNA level of *OCT3/4*. However, there was no association between the mRNA level of the *LGR5* transcript variants and the mRNA levels of *RNF43* and *RSPO1,* which are both directly involved in the LGR5 signalling or the mRNA level of *SNAI1*, an EMT-`mastermind´ (Table 2).

**Table 2:** Correlations between the *LGR5* transcripts mRNA levels and the mRNA levels of different biomarkers

		LGR5FL/RPII	LGR5Δ5/RPII	LGR5Δ8/RPII	LGR5all/RPII
EMTmarkers					
TWIST1	r_s_	0.379	0.258	0.177	0.350
	*p*-value	0.001*	0.031	0.143	0.003
	n	70	70	70	70
SNAI1	r_s_	0.320	0.330	0.228	0.158
	*p*-value	0.007	0.005	0.057	0.191
	n	70	70	70	70
ZEB2	r_s_	0.450	0.456	0.270	0.312
	*p*-value	< 0.0001*	< 0.0001*	0.018	0.006
	n	76	76	76	76
MMP7	r_s_	0.373	0.288	0.308	0.363
	*p*-value	0.001*	0.016	0.01	0.002*
	n	70	70	70	70
TCF7	r_s_	0.581	0.490	0.337	0.553
	*p*-value	< 0.0001*	< 0.0001*	0.004	< 0.0001*
	n	70	70	70	70
Vimentin	r_s_	0.594	0.624	0.454	0.402
	*p*-value	< 0.0001*	< 0.0001*	< 0.0001*	0.001*
	n	70	70	70	70
TGFβ	r_s_	0.402	0.305	0.285	0.261
	*p*-value	< 0.0001*	0.007	0.013	0,023
	n	76	76	76	76
Stem cell markers					
NANOG	r_s_	0.187	0.137	0.219	0.383
	*p*-value	0.121	0.259	0.068	0.001*
	n	70	70	70	70
Oct3/4	r_s_	0.251	0.216	0.377	0.207
	*p*-value	0.036	0.073	0.001*	0.086
	n	70	70	70	70
Proteins involved in the metastatic process					
P4HA1	r_s_	0.390	0.236	0.276	0.167
	*p*-value	< 0.0001*	0.037	0.014	0.144
	n	78	78	78	78
CTGF	r_s_	0.492	0.469	0.302	0.301
	*p*-value	< 0.0001*	< 0.0001*	0.012	0.013
	n	68	68	68	68
IGF2	r_s_	0.498	0.485	0.316	0.271
	*p*-value	< 0.0001*	< 0.0001*	0.009	0.025
	n	68	68	68	68
OPN	r_s_	0.281	0.240	0.301	0.079
	*p*-value	0.020	0.049	0.013	0.522
	n	68	68	68	68
Wnt signaling modulating genes					
RNF43	r_s_	−0.129	−0.171	−0.204	−0.123
	*p*-value	0.287	0.158	0.090	0.311
	n	70	70	70	70
RSPO1	r_s_	0.097	0.131	0.040	0.051
	*p*-value	0.427	0.284	0.743	0.678
	n	69	69	69	69

Bivariable Spearman’s Rho test. r_s_: correlation coefficient. The underlined genes have been shown to be Wnt- target genes. *P*-values were adjusted by the Bonferroni correction.* *p*-value ≤0.003 indicates statistical significance

## Discussion

In this study, we demonstrated that an elevated *LGR5Δ5* mRNA level is an independent negative prognostic marker for overall and disease-specific survival and is associated with the occurrence of regional lymph node metastases in OSCC patients while *LGR5FL, LGR5Δ8* as well as *LGR5all* mRNA levels have no prognostic and predictive impact.

An association between an elevated *LGR5* expression and unfavourable outcome has been reported for several tumor entities. In glioblastoma the number of the *LGR5* expressing cells increased with the tumor staging and correlated with poor outcome [29]. In lung cancer and colon cancer an elevated *LGR5* expression was found to correlate with tumor-size, tumor-stage, metastasis and poor outcome [16, 18, 23, 30]. While in gastric carcinoma, a high *LGR5* expression correlated with lymphatic invasion but not with the risk of regional lymph node metastasis [31]. In the presented study, we found a significant correlation between *LGR5Δ5* mRNA level (*p* = 0.006) and the occurrence of regional lymph node metastases but not for the other LGR5 transcript variants (*LGR5FL* and *LGR5Δ8*) and lymph node involvement in OSCC (Table 1). Furthermore, only the *LGR5Δ5* splice variant but not *LGR5FL or LGR5Δ8* mRNA level have a prognostic value for OSCC patients. In contrast another study in OSCC showed that the LGR5 expression increased during the process of the malignant transformation but there was no association between the LGR5 protein expression and other clinical parameters [22]. However, these investigators performed LGR5 immunohistochemistry and therefore a discrimination between splice variant products of LGR5 was not possible.

Concerning the *LGR5* splice variants only one study analysed the functional difference between *LGR5FL* and the *LGR5* transcript variants *LGR5Δ5* and *LGR5Δ5–8* in respect to cell proliferation. In that study the scientists observed a higher activation of the Wnt signalling together with a higher proliferative ability upon overexpression of both *LGR5* splice variants compared with the cells which overexpressed only *LGR5FL* in colorectal cancer cells [25].

Several studies indicate an association between *LGR5* expression and the expression of other Wnt-target genes, e.g., *ß-catenin* [29, 32–34]. In our study, we found a positive correlation between the mRNA levels of different LGR5 transcript variants and the mRNA levels of Wnt-target genes *MMP7, TCF7, TWIST1*, *Vimentin*, *NANOG*, *OCT3/4* and *ZEB2* was seen*.* Most of these Wnt-target genes are linked to the epithelial-to-mesenchymal transition (EMT). *TWIST* and *ZEB* are the ‘mastermind’ genes of the EMT [35] while *MMP7*, Vimentin*, NANOG*, *OCT3/4* and *TGFß* are also involved in the EMT. According to this finding, for hepatocellular carcinoma it was reported that *LGR5* promotes metastasis through inducting EMT [36]. Moreover, *NANOG* and *OCT3/4* are the key regulators of self-renewal in stem cells [37]*.* Osawa et al. described that the *LGR5FL* expression was restricted to stem cells of the crypts while the expression of the *LGR5* splice variants (*LGR5Δ5* and *LGR5Δ5–8*) was also seen in the middle and the tips of the villi of the small intestine and was associated with a higher proliferative ability [25]. Furthermore, in our study the mRNA level of the *LGR5* transcript variants were positively correlated with the mRNA level of *CTGF, P4HA1,* and *IGF2*, as all of these genes are linked to metastasis in cancer [38, 39]. Altogether, our data show that *LGR5* is strongly associated with an enhanced Wnt signalling pathway and we suggest that the induction of the EMT program could be mediated by *LGR5.*

Although the mRNA expression of genes involved in EMT induction and metastasis is associated with the mRNA expression of all investigated *LGR5* transcript variants in this study, only OSCC patients with a high tumoral *LGR5Δ5* level have a significant higher risk of regional lymph node metastasis. We hypothesize that this finding might be due to the induction of the EMT program combined with a higher proliferative ability [25] of the *LGR5Δ5* overexpressing tumor cell, which results in a faster tumor growth and progression and leads to a shorter overall survival of OSCC patients.

## Conclusion

The data presented in this study show that an elevated mRNA level of the *LGR5* splice variant *LGR5Δ5* is an independent negative prognostic factor for OSCC patients as well as correlates with the risk of lymphatic metastasis. Moreover, we suggest that *LGR5* is involved in the EMT process and postulate that this happens predominantly through the activation of the Wnt signalling. Thus our results indicate that *LGR5* might be involved in tumor progression and metastasis of OSCCs. An elevated expression of the *LGR5* splice variant *LGR5Δ5* could be used as a potential prognostic biomarker marking an unfavorable prognosis but has to be analyzed in prospective studies for its application as therapeutic biomarker in OSCC patients.

## Abbreviations

AUC: Area under the curve
CSC: Cancer stem cell, ROC, Receiver operating characteristic
CTGF: Connective tissue growth factor
EMT: Epithelial to Mesenchymal Transition
HR: Hazard ratio; 95% Cl, 95% confidence interval
IGF2: Insulin like growth factor 2
LGR5: Leucine-rich repeat-containing G protein-coupled receptor
MMP7: Matrix metallopeptidase 7
NANOG: Nanog homeobox
Oct3/4: POU class 5 homeobox 1
OPN: Osteopontin
OSCC: Oral squamous cell carcinoma
P: Probability
P4HA1: Prolyl 4-hydroxylase subunit alpha 1
SNAI1: Snail family transcriptional repressor 1
TCF7: Transcription factor 7
TGFβ: Transforming growth factor beta 1
TWIST1: Twist family bHLH transcription factor 1
ZEB2: Zinc finger E-box binding homeobox 2
